# Echocardiographic Abnormalities in Autosomal Dominant Polycystic Kidney Disease (ADPKD) Patients

**DOI:** 10.3390/jcm11205982

**Published:** 2022-10-11

**Authors:** Mariana Becker Pfeferman, Daniel Ribeiro da Rocha, Fernanda Guedes Rodrigues, Elcio Pfeferman, Ita Pfeferman Heilberg

**Affiliations:** 1 Universidade Santo Amaro (UNISA) Medical School, São Paulo 04829-300, Brazil; 2 Nephrology Division, Universidade Federal de São Paulo (UNIFESP), São Paulo 04023-062, Brazil; 3 Nutrition Post Graduation Program, Universidade Federal de São Paulo (UNIFESP), São Paulo 04023-062, Brazil; 4 Hospital Israelita Albert Einstein (HIAE), São Paulo 05652-900, Brazil

**Keywords:** ADPKD, cardiovascular, echocardiogram, left ventricular mass index, mitral valve prolapse, polycystic disease, valvar abnormalities

## Abstract

Cardiovascular abnormalities, such as left ventricular hypertrophy and valvular disorders, particularly mitral valve prolapse, have been described as highly prevalent among adult patients with autosomal dominant polycystic kidney disease (ADPKD). The present study aimed to assess echocardiographic parameters in a large sample of both normotensive and hypertensive ADPKD patients, regardless of kidney function level, and evaluate their association with clinical and laboratorial parameters. A retrospective study consisted of the analysis of clinical, laboratorial, and transthoracic echocardiograms data retrieved from the medical records of young adult ADPKD outpatients. A total of 294 patients (120 M/174 F, 41.0 ± 13.8 years old, 199 hypertensive and 95 normotensive) with a median estimated glomerular filtration rate (eGFR) of 75.5 mL/min/1.73 m^2^ were included. The hypertensive group (67.6%) was significantly older and exhibited significantly lower eGFR than the normotensive one. Increased left ventricular mass index (LVMI) was seen in 2.0%, mitral valve prolapse was observed in 3.4%, mitral valve regurgitation in 15.3%, tricuspid valve regurgitation in 16.0%, and aortic valve regurgitation in 4.8% of the whole sample. The present study suggested that the prevalence of mitral valve prolapse was much lower than previously reported, and increased LVMI was not seen in most adult ADPKD patients.

## 1. Introduction

Autosomal dominant polycystic kidney disease (ADPKD) is one of the most common monogenic kidney diseases, with an estimated prevalence between 1 to 2500 through 1 to 1000 individuals [1,2]. ADPKD is characterized by the formation of cysts along the entire nephron, which increase progressively in number and size, cause architectural destruction of the kidney, triggering a gradual loss of function terminating in chronic kidney disease (CKD) [3]. End-stage chronic kidney disease (ESKD) typically occurs in 50% of patients around the 6th decade of life and corresponds to 5 to 10% of patients under renal replacement therapy [4]. Arterial hypertension (AH), an extrarenal non-cystic manifestation frequently seen in ADPKD is derived from compression of the parenchyma and vasculature by cystic growth, activating renin angiotensin-aldosterone system (RAAS) and renal sympathetic nervous system, causing intrarenal ischemia [5,6]. Reduced exercise-induced vasodilation capacity due to lower systemic nitric oxide levels [7] and non-dipping pattern in ambulatory blood pressure monitoring (ABPM) [8,9], indicating endothelial dysfunction, suggest a future risk of developing hypertension in ADPKD [10]. Early onset AH and the increase in total kidney volume (TKV) are some of the most important surrogate prognostic biomarkers of the progression of the disease [10,11]. Beyond AH and intracranial aneurysms, other cardiovascular abnormalities, such as left ventricular hypertrophy, arrhythmias, valvular disorders, and dilated cardiomyopathy have been described [12,13,14,15], all of them representing potential players in morbidity and mortality in this population [12]. The high incidence of cardiovascular complications may be explained not only by precocious manifestation of hypertension [13] since normotensive ADPKD may also present higher left ventricular mass index (LVMI), dilated cardiomyopathies, and biventricular diastolic dysfunction [16,17]. Moreover, given that polycystins exhibit an important role in heart embryogenesis [18,19] and Pkd1-deficient non-cystic mice already show cardiac dysfunction [20], a primary genetic effect of the dysregulation of polycystin function on cardiovascular abnormalities in ADPKD may exist. Several echocardiographic studies in the last decades have been conducted on different populations, providing some controversial data. The present study aimed to assess cardiac structure and function by echocardiography in a large sample of both normotensive and hypertensive ADPKD patients, regardless of their level of renal function, and evaluate their association with clinical and laboratorial parameters.

## 2. Materials and Methods

### 2.1. Study Design and Participants

This is a retrospective study based on the medical records of ADPKD patients followed at the outpatient Polycystic Kidney Disease (PKD) Unit of the Nephrology Division of the Universidade Federal de São Paulo (UNIFESP) from March 2002 through June 2018, whose transthoracic echocardiograms (TTE) were available as part of their routine evaluation. Exclusion criteria were age < 18 years, malignant diseases, and ESKD on dialysis. The diagnosis of ADPKD was confirmed by positive family history and renal cysts according to ultrasonographic diagnostic criteria by Pei et al. [21].

### 2.2. Ethical Considerations 

The study was reviewed and approved by the Research Ethics Advisory Committee of the Universidade Federal de São Paulo (UNIFESP) (CAAE: 01521418.2.0000.5505).

### 2.3. Data Collection

Demographic, anthropometric, and serum biochemistry data were retrieved from patients’ electronic records. Biochemical parameters considered for the present analysis were serum creatinine, sodium, potassium, uric acid, fasting glucose, low-density lipoprotein (LDL) cholesterol, high-density lipoprotein cholesterol (HDL), and triglycerides. Creatinine was determined by an isotope dilution mass spectrometry (IDMS) traceable method and the estimated Glomerular Filtration Rate (eGFR) was calculated using the CKD-EPI equation. Total Kidney Volume (TKV) was calculated and defined as the mean of both kidneys based on renal ultrasound using a standard formula of a modified ellipsoid for each kidney as follows: Renal volume = 4/3 π (anteroposterior diameter/4 + width/4)^2^ × length/2. Hypertension at admission was defined based on office or clinic levels of systolic blood pressure ≥ 140 mmHg or diastolic blood pressure ≥ 90 mmHg [22] or use of antihypertensive medications. Co-morbidities, such as dyslipidemia, diabetes mellitus, previous acute myocardial infarction (AMI) or coronary artery disease (CAD), stroke, and tobacco use, were also included in the analysis. 

Transthoracic echocardiograms (TTE) was performed on a Philips IE33 echocardiography device (Andover, MA, USA) using a S5–1 (1–5 mHz) transducer, capable of obtaining two-dimensional images and analysis of the flow velocity with color and flow mapping techniques and Spectral Doppler. The measurements were obtained as recommended by the American Society of Echocardiography [23] and the Left Ventricle (LV) ejection fraction (EF) was calculated using the modified Simpson’s method. The following echocardiographic parameters were considered for the present analysis: Aortic root diameter (AO) and left atrium (LA) volume, left ventricle (LV) posterior wall (LVPW) thickness, interventricular septum (IVS) thickness, LV ejection fraction (EF) and LV Mass Index (LVMI). The upper limits of normality for LVMI were considered to be 135 g/m^2^ (males) and 111 g/m^2^ (females). Valvular disorders included Mitral, Aortic and Tricuspid Valve Regurgitation, Mitral Valve Prolapse (MVP), and Diastolic function. Based on the 2003-Guideline from the American Society of Echocardiography [24], the severity of regurgitation was assessed by doppler echocardiography and grades were classified into mild, moderate, and severe. For the purpose of the present analysis, minimal (trace or trivial) regurgitation was not considered since it can be barely detected and is usually physiologic. For mitral and tricuspid regurgitation, three methods were used to quantify severity: Regurgitant jet area, vena contracta, and flow convergence (PISA), which provides EROA (effective regurgitant orifice area). For aortic regurgitation, the assessment was based on the proximal jet width or cross-sectional area immediately below the aortic valve, within 1 cm of the valve. Mitral valve prolapse was diagnosed as systolic displacement of mitral leaflet into the LA of at least 2 mm from the mitral annular plane [25].

### 2.4. Statistical Analysis

Statistical analyses were performed using IBM SPSS version 23.0 (SPSS Inc., Chicago, IL, USA). In all analyses, *p* < 0.05 was considered significant. Variable distributions were evaluated by the Kolmogorov–Smirnov test. Normally distributed variables were expressed as mean ± standard deviation (SD) and non-normally distributed ones as median (interquartile range). Differences between groups were tested by Mann–Whitney U pairwise or Student t-test according to their distribution. Categorical variables, presented as n (%), were compared using a Chi-square test. A logistic binary regression using left ventricle impaired relaxation (yes or no) as a dependent variable, and a multivariable linear regression using LVMI as a dependent variable, were additionally performed.

## 3. Results

### 3.1. Populations Demographic and Clinical Characteristics 

From a total of 557 ADPKD patients referred for care at the outpatient PKD unit, TTE data were available for 346. Of these, 52 did not meet inclusion criteria, resulting in 294 subjects for the present analysis. Table 1 summarizes clinical and demographic characteristics of the whole sample as well as according to their classification as hypertensive (n = 199, 67.6%) or normotensive patients (n = 95, 32.4%). Females were predominant in the whole sample (59.2%) as well as Caucasians (61.6%) and mean age was 41.0 ± 13.8 years old. The hypertensive group was significantly older, exhibited significantly higher mean BMI, median TKV, median kidney length and presented with significantly higher rates of dyslipidemia and diabetes than the normotensive group. The percentage of patients with history of AMI, CAD, or stroke did not differ between groups. Within the normotensive group, most patients belonged to stages I, II, or III of CKD, (75.8%, 20.0%, and 4.2%, respectively), contrasting with fewer patients in such classes in the hypertensive group (16.6%, 30.1%, and 33.2%, respectively), with statistically significant differences. CKD stages IV and V were disclosed only among hypertensive patients (16.1% and 4.0%, respectively). The use of tobacco in the whole sample was observed in 13.6%, with no significant differences between hypertensive or normotensive groups. The majority of hypertensive patients used ACEi or ARB (88.9%), diuretics (53.8%), followed by calcium channel blockers or β-blockers, alone or in association (Table 1). None of the normotensive patients used ACEi or ARB.

### 3.2. Laboratory and Echocardiographic Findings 

Table 2 shows the laboratorial and echocardiographic parameters from all patients, also according to the presence or not of hypertension. The hypertensive group presented significantly lower eGFR, higher serum sodium, potassium, uric acid, fasting glucose LDL and triglycerides. With respect to echocardiographic parameters, hypertensive patients exhibited significantly higher mean values of AO and LA diameters, IVS, LVPW thickness, and LVMI than the normotensive group. Only 6 out of 294 patients (2.0%) presented LVMI values above the upper limit of normality. The results of a linear regression using LVMI as a dependent variable are shown in Table 3. In the multivariate model, age and sex were the only independent determinants of LVMI.

A significantly higher mean percentage of patients with impaired LV relaxation was noticed in the hypertensive versus normotensive groups (35.2 vs. 6.3%, *p* < 0.001), but hypertension was no longer associated with an impaired LV relaxation when performing a logistic binary regression analysis adjusted for age, BMI and eGFR (HR 0.47 [95% CI 0.20–1.23], *p* = 0.09).

### 3.3. Valvar Abnormalities 

**Figure 1:**
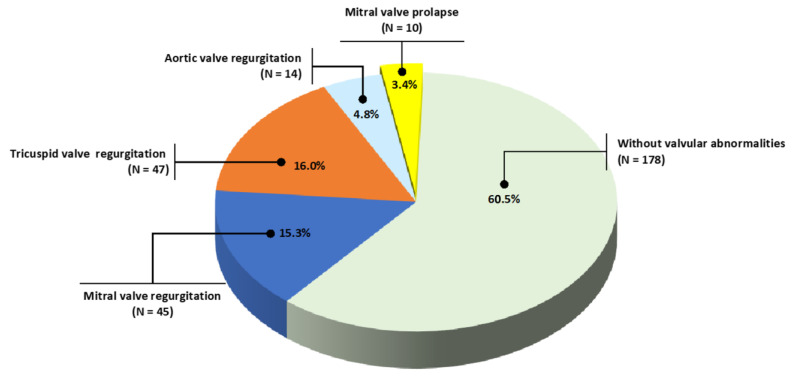
Valvar abnormalities.

As shown in Figure 1, mitral valve prolapse (MVP) was present in only 3.4% of patients and mitral, aortic, and tricuspid, valve regurgitation in 15.3%, 4.8% and 16.0% of patients. There were no statistical differences between hypertensive or normotensive groups for mitral regurgitation (17.6% vs. 10.5%, respectively, *p* = 0.08), and tricuspid regurgitation (16.6% vs. 14.7%, respectively, *p* = 0.41). Aortic regurgitation was significantly higher among hypertensives (6.5 vs. 1.0%, *p* = 0.03), whereas MVP was higher among normotensives (8.4 vs. 1.0%, *p* < 0.001).

## 4. Discussion

Structural and functional cardiovascular alterations, particularly those detected by echocardiography, have been described to be associated with ADPKD [12,13,26,27] and may be important contributors to greater morbidity and mortality in this population [28,29]. However, previous echocardiographic data deriving from most studies conducted between 1980 and 2000 on distinct populations employed different methodologies, yielding heterogeneous results. Herein, we retrospectively assessed echocardiographic parameters and searched for cardiac valvar abnormalities in a large sample of adult patients with ADPKD. Our main findings were a lower prevalence of increased LVMI and valvar lesions than the ones described in most series of adult ADPKD patients, especially concerning mitral valve prolapse (MVP).

Present rates of 3.4% of MVP are similar to the ones described in the general population, around 2.5–3.0% [30,31,32,33,34] but are in contrast with previous rates observed in ADPKD patients ranging from 12% up to 26% in some studies [13,35,36,37,38]. Conversely, some investigators reported rates of 9% for MVP [39], while others had not seen any case of MVP in their sample [40] nor any other cardiac valvar defect at all [41]. In line with the current series, a very recent contemporary cohort of children and young people screened for MVP by Savis et al. [42] showed a prevalence of 0.98% of MVP, without any case of mitral regurgitation. These investigators found variations in normal valvar anatomy in 8.8% of patients, not fulfilling the criteria of true MVP. All this controversy might have accounted for changes in the techniques to evaluate MVP, which were altered in the late 1980s [43,44], or to the redefinition of criteria for echocardiographic parameters dating from the end of the 1990s [33,34]. Therefore, leaflets that appeared to be prolapsed were later found to be normal. The greater part of data investigating valvopathies in ADPKD arising from that period, employed the M-mode echocardiographic method to identify MVP. Subsequently, such technique was shown to fail in displaying the leaflets in relation to their surrounding annular attachments [45], and the results varied widely depending on the orientation of the transducer [33,46]. A study that compared M-mode, two-dimensional, and doppler echocardiography showed that the sensitivity of bidimensional method was significantly higher than M-mode [47]. The association of MVP with age and hypertension in the ADPKD setting is also under debate with some authors finding MVP related to higher blood pressure in children [38], whereas others reported its decrease with age and lack of any association with hypertension [35]. In the current series, MVP is even less prevalent in hypertensive than normotensive ADPKD patients.

Mitral valve regurgitation was disclosed in 15.3% of the present sample, with no differences between hypertensive and normotensive patients contrasting with the description of up to 31% of ADPKD patients in the literature [36,37]. Despite the difficulty of determining the frequency of this alteration in presumed healthy subjects, Choong et al. [48] described a prevalence of 34% of mitral valve regurgitation in the general population. However, when disregarding minimal grades of mitral valve regurgitation, as we did in our sample, their percentage drops by more than half. Minimal grades of regurgitation in any cardiac valve are recognized to be a normal variant and can occur even when the valvular morphology and leaflet motion are normal [49]. Among patients with ADPKD, Hossack et al. [36] demonstrated mitral valve regurgitation in 31% of cases, but it is noteworthy that these investigators also included minimal mitral valve regurgitation, which represented more than 20% of cases, leading the actual prevalence to decrease to approximately 10%. Lumiaho et al. [35], on the other hand, showed a prevalence of 13% of mitral valve regurgitation in ADPKD, being all the regurgitation grades 2 and 3. When dividing their population according to the presence of hypertension and level of renal function, they observed a significant increase in the frequency of mitral valve regurgitation in hypertensive individuals and individuals with worse renal function, which was not observed in our study (*p* = 0.08). Interestingly, a very recent small retrospective study with patients who underwent echocardiography and whose genotype was confirmed by genetic testing showed a higher prevalence of mitral valve regurgitation in patients with PKD-1 genotype than in those with PKD-2 or non-PKD-1 (46.9% vs. 8.3% vs. 19.0%), with no other cardiac valve complications [50].

The prevalence of 4.8% of aortic valve regurgitation disclosed in our sample, more common among the hypertensive group, was nevertheless lower than the one reported in the general population of similar age (9%) [48] and also if compared to ADPKD patients, (8% for grades 2 or 3) [35]. In contrast to the present and previous studies, Leier et al. [39] reported a higher prevalence of aortic valve regurgitation than mitral valve regurgitation in a small series of 11 ADPKD patients, evidencing once again the heterogeneity of echocardiographic results. Noteworthy, advanced stages CKD may be accompanied by valve annulus calcification, especially in mitral and aortic valves [51,52,53], but in the present sample, there have been only 4.0% of patients in CKD stage V.

Finally, we observed tricuspid valve regurgitation in 16% of patients (not differing statistically in the presence of hypertension), in line with rates reported by Hossack et al. [36] (15%), but higher than those by Lumiaho et al. [35] (4%). Present percentages were lower than the ones described by healthy individuals of the same age (22%) [48]. The reasons for these differences remain unexplained, but according to Choong et al. [48], valvular regurgitation is not uncommon in structurally normal hearts being mitral and tricuspid the most frequent ones, and as the prevalence increases with aging, a wear-and-tear phenomenon must be balanced against a congenital cause. However, trivial tricuspid regurgitation, which represents more than 10% of their sample, was not included in the present study.

Given that the pathophysiology and epidemiology of cardiac valvar abnormalities, particularly MVP, remain not fully understood even in the general population [54], the underlying mechanisms by which these abnormalities arise in ADPKD, remain even less clear. Additional experimental studies and the advent of newer technologies to carefully measure mitral valve function are still warranted to elucidate if MVP and other valvar disorders are still important extrarenal manifestations in ADPKD.

In the present study, the dimensions of the cardiac chambers and aorta, as well as parameters related to myocardial mass, were within limits established by the American Society of Echocardiography Guidelines, elaborated in conjunction with the European Association of Echocardiography [23]. The majority of measurements in our sample were similar to a report of a Brazilian cohort of asymptomatic individuals, with no history of cardiovascular disease, with the same age and sex distribution, as reported by Ângelo et al. [55]. The present mean values of AO, LA, IVS thickness, LVPW and LVMI were statistically higher for the hypertensive group, although still within the normal range (Table 1). However, when performing a multivariable linear regression analysis, hypertension was not an independent determinant of LVMI (*p* = 0.09). Of note, when we compared the mean values of LVMI between our normotensive patients with the cohort of healthy individuals reported by Ângelo et al. [55], there were no statistical differences in the groups aged 30–39 years old (79.0 ± 13.5, n = 20 vs. 75.1 ± 14.3, n = 57, *p* = 0.28) and aged 40–49 years old (72.6 ± 16.5, n = 12 vs. 77.9 ± 13.1, n = 39, *p* = 0.33), respectively. Moreover, only 2.0% of the present cohort exhibited increased LVMI. Left ventricular hypertrophy (LVH) is highly frequent among pediatric and adult patients with ADPKD [13,16,26,38,41,56,57] associated with hypertension [38,57] or not [8,41,56,58]. According to some studies, increased LVMI in normotensive ADPKD patients correlated with 24h ambulatory systolic BP or with an exaggerated systolic BP response to exercise [8,59]. However, the prevalence of increased LVMI is highly variable in the literature ranging from up to 48% in older series [26] down to 20% or less in more recent reports [60]. Noteworthy, the percentage of increased LVMI measured by magnetic resonance imaging (a gold standard method for assessing LV mass) [14] in the HALT-PKD study [61] involving 558 hypertensive ADPKD patients under pharmacological therapy showed a prevalence of left ventricular hypertrophy (LVH) as low as 0.93%. Potential contributors to the lower LVMI values in this large trial might have been the different imaging technique, an earlier diagnosis of hypertension with more rigorous control of BP, and use of renin–angiotensin–aldosterone system inhibitors by more than 80% [13,14,62], as seems to have been the case in our series as well.

Beyond the impact of hypertension on echocardiographic alterations in the current study, the hypertensive sub-group was also significantly older, presented higher BMI, TKV and kidney length, more diabetes or features of metabolic syndrome (hyperuricemia, dyslipidemia), and lower kidney function when compared to the normotensive group, suggesting a longer duration of the disease. A recent review of LVH beyond hypertension in ADPKD showed that there are factors other than hypertension that contribute to LVH [63]. Insulin resistance has been associated with LVMI in ADPKD patients independently of age, weight, BP, and albuminuria [64]. The Chronic Renal Insufficiency Cohort (CRIC) Study [65] showed an association between the degree of loss of renal function and abnormalities in cardiac structure, mainly higher left ventricular mass, but polycystic kidney disease had been excluded from the trial. Chen et al. [60] observed an inverse correlation between eGFR and LVMI. In the present series, eGFR was not an independent determinant of LVMI by multivariable linear regression. Park et al. [66] revealed changes in ventricular structure, with a prevalence of 57% of LVH in CKD patients with an eGFR < 45 mL/min/1.73 m² and 32% for those with eGFR > 60 mL/min/1.73 m², but with no evidence of systolic or diastolic dysfunction. Therefore, it is possible that CKD itself, regardless of ADPKD as the underlying disease, is responsible for such findings. Moreover, Martinez-Vea et al. [67] compared echocardiographic parameters of dialysis patients with ADPKD with patients with CKD from other underlying kidney diseases and did not observe any significant difference between the two groups, reinforcing once more that the observed changes might have resulted from decreased renal function and not from ADPKD.

The prevalence of impaired left ventricular relaxation of 25.8% in the present series suggesting diastolic dysfunction, much higher than observed in the general population (11%) [53], was statistically higher among the hypertensive group. However, as aforementioned, the hypertensive group was older and had less preserved kidney function. Nonetheless, according to Otsuka et al. [68], the change in relaxation can be observed even in the early stages of kidney dysfunction. Moreover, other investigators [16,38,56] observed that LV diastolic function was also impaired among normotensive ADPKD patients and was not dependent on age [27]. On the other hand, when using tissue Doppler imaging (TDI) techniques, diastolic dysfunction was not evidenced in young normotensive ADPKD patients [58]. Of note, hypertension was no longer associated with an impaired LV relaxation in our current series when performing a logistic binary regression analysis adjusted for age, BMI and eGFR (*p* = 0.09).

It seems that more accurate assessment of cardiac diastolic function, such as TDI, spectral Doppler, and speckle strain echocardiography are still needed (especially when ejection fraction is preserved) as more appropriate surrogate markers of diastolic assessment as opposed to a single parameter measurement [69]

Limitations of our study include the characteristic observational and retrospective design, the lack of a control group, no PKD1 or PKD2 genotyping, and determination of kidney volume by ultrasound. However, its strength relies on the larger sample size when compared to the previous reports.

## 5. Conclusions

In conclusion, the present study disclosed a lower prevalence of mitral valve prolapse and did not show increased LVMI in adult ADPKD patients contrasting with previous reports. Further prospective studies on other populations are warranted to confirm these findings.

## Figures and Tables

**Table 1:** Demographic and clinical characteristics in ADPKD patients.

	Total (n = 294)	Hypertensive (n = 199)	Normotensive (n = 95)	*p* Value
Sex (Male)	120 (40.8)	79 (39.7)	41 (43.2)	0.33
Age (years)	41.0 ± 13.8	46.0 ± 12.1	30.0 ± 10.9	<0.01
BMI (kg/m^2^)	25.4 ± 4.6	26.5 ± 4.6	23.3 ± 3.6	<0.01
Race				
Caucasian	181 (61.6)	121 (60.8)	60 (63.1)	0.87
Non-caucasian	113 (38.4)	78 (39.2)	35 (36.7)	
Dyslipidemia	46 (15.6)	42 (21.1)	4 (4.2)	<0.01
Diabetes mellitus	9 (3.0)	9 (4.5)	0 (0)	0.03
AMI/CAD	1 (0.3)	1 (0.5)	0 (0)	0.67
Stroke	5 (1.7)	5 (2.5)	0 (0)	0.14
CKD stages				
CKD I	105 (35.7)	33 (16.6)	72 (75.8)	<0.001
CKD II	79 (26.9)	60 (30.1)	19 (20.0)	
CKD III	70 (23.8)	66 (33.2)	4 (4.2)	
CKD IV	32 (10.9)	32 (16.1)	0 (0)	
CKD V	8 (2.7)	8 (4.0)	0 (0)	
TKV (mL) *	476.6 (236.2–915.6)	679.2 (371.1–1124.7)	237.6 (171.3–375.8)	<0.01
Kidney Length (cm) *	16.9 (13.4–23.3)	20.3 (15.6–25.6)	13.6 (12.0–16.1)	<0.01
Tobacco use	40 (13.6)	31 (15.6)	9 (9.4)	0.11
Antihypertensive drugs				
ACEi/ARB	177 (88.9)	177 (88.9)	-	-
Diuretics	107 (53.8)	107 (53.8)	-	-
Calcium channel blockers	54 (27.1)	54 (27.1)	-	-
Beta-blockers	44 (22.1)	44 (22.1)	-	-

Data as n (%), mean ± standard deviation (SD) or median (interquartile interval), BMI (body mass index); AMI (acute myocardial infarction)/CAD (coronary artery disease); CKD (chronic kidney disease), TKV (total kidney volume) ACEi (ACE inhibitors; ARB (Angiotensin II Receptor Blockers) * Missing data: 79.

**Table 2:** Laboratory and echocardiographic parameters in ADPKD patients.

	Total (n = 294)	Hypertensive (n = 199)	Normotensive (n = 95)	*p* Value
sCreatinine (mg/dL)	1.08 (0.86–1.57)	1.29 (0.98–1.92)	0.89 (0.76–1.00)	<0.01
eGFR (mL/min/1.73 m^2^)	75.5 (44.5–101.0)	56.5 (34.1–81.6)	105.2 (90.1–115.7)	<0.01
sNa (mEq/L)	140.0 (138.0–142.8)	141.0 (138.8–143.0)	139.0 (137.0–142.0)	<0.01
sK (mEq/L)	4.4 (4.1–4.7)	4.4 (4.1–4.8)	4.25 (4.0–4.6)	0.02
sUric Acid (mg/dL)	5.6 (4.6–7.1)	6.2 (5.0–7.6)	4.6 (3.9–5.5)	<0.01
sGlucose (mg/dL)	92.0 (87.0–98.0)	94.0 (88.0–100.0)	88.0 (84.5–95.0)	<0.01
HDL (mg/dL)	49.0 (41.0–57.0)	48.5 (39.8–55.3)	50.0 (42.0–60.5)	0.14
LDL (mg/dL)	108.0 (88.0–128.0)	111.0 (93.0–135.0)	95.5 (75.8–116.0)	<0.01
Triglycerides (mg/dL)	104.0 (75.0–151.0)	123.5 (86.8–166.3)	82.0 (62.0–104.5)	<0.01
Echocardiographic parameters				
AORTA (mm)	31.0 (30.0–34.0)	32.0 (30.0–35.0)	30.0 (28.0–32.0)	<0.01
LA (mm)	35.0 (32.0–37.0)	36.0 (34.0–38.0)	34.0 (30.0–36.0)	<0.01
IVS (mm)	9.0 (8.0–10.0)	9.0 (9.0–10.0)	8.0 (8.0–9.0)	<0.01
LVPW (mm)	9.0 (8.0–9.0)	9.0 (8.0–10.0)	8.0 (8.0–9.0)	<0.01
LVMI (g/m^2^)	78.8 (69.7–90.5)	81.8 (72.3–91.9)	75.8 (64.4–84.9)	<0.01
Increased LVMI—n (%)	6 (2.0)	6 (2.0)	0 (0)	
EF (%)	68.0 (65.0–71.0)	68.0 (66.0–71.0)	67.5 (64.0–70.0)	0.23
Impaired LV relaxation—n (%)	76 (25.8)	70 (35.2)	6 (6.3)	<0.001

Data expressed in mean (interquartile interval) or number and percentage of patients n (%). Abbreviations: eGFR (estimated Glomerular Filtration Rate) Na (sodium), K (potassium), HDL (high density lipoprotein), LDL (low density lipoprotein), LA (left atrium), IVS (interventricular septum), LVPW (Left Ventricular Posterior Wall) LVMI (Left Ventricular Mass Index), EF (Ejection Fraction), LV (left ventricular).

**Table 3:** Possible determinants of LVMI using linear regression.

	LVMI			
Independent Variables	Univariate		Multivariate *	
	β	*p*	β	*p*
Age (years)	0.25	<0.01	0.22	<0.01
Sex, (M)	0.15	0.02	0.19	<0.01
BMI (kg/m^2^)	0.16	0.01	-	-
Tobacco use (yes)	0.15	0.02	-	-
Hypertension (yes)	0.23	<0.01	0.12	0.09
Dyslipidemia (yes)	0.07	0.26	-	-
eGFR (mL/min/1.73 m^2^)	−0.18	<0.01	-	-
Uric Acid (mg/dL)	0.11	0.14	-	-
LDL (mg/dL)	0.02	0.74	-	-
Triglycerides (mg/dL)	0.18	0.01	-	-
ACEi/ARB (yes)	0.04	0.58	-	-

Abbreviations: LVMI (Left Ventricular Mass Index), BMI (Body Mass Index), eGFR (estimated Glomerular Filtration Rate), LDL (low density lipoprotein), ACEi (ACE inhibitors), ARB (Angiotensin II Receptor Blockers). * Run backwards.

## Data Availability

Data presented in this study will be provided upon reasonable request.
